# Pre-match Warm-Up Dynamics and Workload in Elite Futsal

**DOI:** 10.3389/fpsyg.2020.584602

**Published:** 2020-11-26

**Authors:** Nuno Silva, Bruno Travassos, Bruno Gonçalves, João Brito, Eduardo Abade

**Affiliations:** ^1^Research in Sports Sciences, Health Sciences and Human Development, CIDESD, University Institute of Maia, ISMAI, Maia, Portugal; ^2^Department of Sports Sciences, Research Center in Sports Sciences, Health Sciences and Human Development (CIDESD), University of Beira Interior, Covilhã, Portugal; ^3^Portugal Football School, Portuguese Football Federation, Oeiras, Portugal; ^4^Departamento de Desporto e Saúde, Escola de Ciências e Tecnologia, Universidade de Évora, Évora, Portugal; ^5^Comprehensive Health Research Centre (CHRC), Universidade de Évora, Évora, Portugal

**Keywords:** potentiation, performance enhancement, priming methods, current practices, warm-up structure

## Abstract

Warming up prior to competition is a widely accepted strategy to increase players’ readiness and achieve high performances. However, pre-match routines are commonly based on empirical knowledge and strongly influenced by models emerging from elite team practices. The aim of the present study was to identify and analyze current pre-match warm-up practices in elite futsal. Forty-three elite players were analyzed during their pre-match warm-up routines during the Portuguese Futsal Cup Final 8. Warm-up tasks were classified according to duration, type of activity, sequence, and structure. External load variables included the total distance covered, total distance covered per minute, running distance per minute, sprinting distance per minute, number of accelerations per minute, and number of decelerations per minute. Results highlighted that warm-up routines lasted for 27.5 ± 9.2 min and included nine major different tasks. Open-skill activities were prioritized by coaches; competitive and non-competitive futsal-specific tasks were included in 90% of the total warm-up routines, with higher focus on non-competitive tasks (68% of total time). The intensity progressively increased during warm-ups, mainly because of the higher number of accelerations and decelerations per minute. Pre-match warm-up routines strongly endorse futsal-specific and representative tasks in order to establish pre-match settings able to prepare players for the upcoming game. When designing pre-match warm-up routines, coaches should be aware that duration, sequence, and type of tasks may affect players’ acute performance and readiness.

## Introduction

Futsal is a complex and dynamic team sport requiring players to combine decision-making processes and intermittent high-intensity actions such as sprints and changes of direction (Barbero-Alvarez et al., 2008). Futsal players cover more than 25% of the distance at high speed or sprinting with a work-to-rest ratio of 1:1 (Barbero-Alvarez et al., 2008) and repeated sprint blocks of two to three sprints interspersed by 15-s intervals (Caetano et al., 2015). Pre-match warm-up plays an important role in order to help players to cope with these demands and support acute performance potentiation. In fact, warm-up routines are known to promote temperature, metabolic, psychological, and neuromuscular mechanisms (McGowan et al., 2015) that may be key to improve players’ readiness and potentiate neuromuscular performance before training or competition.

Under the scope of performance potentiation, neuromuscular function appears to be acutely increased when preceded by maximal or submaximal efforts (Wilson et al., 2013), a phenomenon known as post-activation potentiation (PAP). PAP is strongly related to the enhanced central activity of the motor neurons (Tillin and Bishop, 2009), spinal cord reflex activity, and phosphorylation of the myosin chain (Smith and Fry, 2007), which leads to an increase in the sensitivity of the myofilaments to Ca2^+^ (MacIntosh, 2003). Recently, a new concept of PAP has been proposed in order to be more convergent with the timeline of peak voluntary performance enhancement. Thus, post-activation performance enhancement (PAPE) occurs when a high-intensity voluntary conditioning contraction leads to enhancement of subsequent involuntary muscular performance without confirmatory evidence of classical PAP (Cuenca-Fernandez et al., 2017).

Post-activation performance enhancement effect can be explained with the increase of muscle temperature, fiber water content, and activation, but inhibited by residual fatigue and motor pattern interference (Blazevich and Babault, 2019), which means that its effectiveness depends on the balance between potentiation and fatigue (Tillin and Bishop, 2009). The mechanisms underlying the warm-up are well aligned with the PAPE effects and should be taken in consideration when designing a warm-up routine.

Active warm-up routines are commonly used as a strategy for pre-competition preparation in team sports, generally including a wide variety of closed and open skills to improve key abilities such as speed, change of direction speed, vertical jump, and reactive agility performance (Gabbett et al., 2008). Under this scope, exercises such as small-sided games (SSG) and shooting exercises are often proposed by coaches. These exercises can potentially boost performance through priming neural pathways and increased neuromuscular activation while maintaining a link to technical and tactical components (McGowan et al., 2015). Other activities such as sprinting and stretching are also commonly used in warm-up routines (Ayala et al., 2012). Nevertheless, a stronger consensus is missing regarding the effects, moment, and type of sprinting and stretching (Guinoubi et al., 2015; van den Tillaar and von Heimburg, 2016).

When designing a warm-up routine, coaches should take into consideration several factors such as duration and total volume, intensity, sequence, and the dynamics of the selected exercises. In team sports, the warm-up is frequently performed over an extended period of time (>20 min), which may promote fatigue and inhibit performance enhancements (Silva et al., 2018). Even though a shorter warm-up appear to have similar benefits as a longer warm-up (Mujika et al., 2012; van den Tillaar and von Heimburg, 2016), some players report the need for longer warm-up periods to feel psychologically prepared for competition (Yanci et al., 2019).

Regardless of exercise sequence, the warm-up should progress in intensity, preparing for the specific tasks of the sport, and finishing with tasks of maximum intensity (Silva et al., 2018). However, it is also suggested that short-term explosive exercises such as sprinting and jumping may benefit from high-intensity warming up with an appropriate recovery so that the athlete is able to produce high levels of strength early in a team sports game (Anderson et al., 2014). Thus, the main challenge seems to be the identification of appropriate exercises and sequence capable of promoting the ideal intensity and recovery.

Despite the importance of structure and dynamics of warm-up in team sports, exercises are still being selected based on empirical knowledge and might be strongly influenced by observations on routines conducted by high-level teams. Thus, understanding warm-up routines in elite teams might provide key hints to develop evidence-based warm-up routines with great ecological value. To the best of our knowledge, and despite some theoretical proposals, there is no research focused on the analysis of warm-up structure and intensity in futsal. Thus, the aims of this study were to identify current pre-match warm-up practices in elite futsal teams and to analyze its dynamics and workload.

## Materials and Methods

### Participants

Forty-three elite male futsal players from six different futsal elite teams participated in this study. Data were collected during a futsal major event—Final 8 of the Portuguese Futsal Cup. The event lasted for 4 days during the in-season period (May 2019). Goalkeeper (GK) data were excluded due to the specificity of goalkeeping role and warm-up routine. All players, coaches, and clubs were informed about the study design, requirements, and procedures, and their consent was obtained. The study protocol was approved by the local Ethics Committee of Universidade da Beira Interior (CE-UBI-Pj-2018-029) and follows the recommendations of the Declaration of Helsinki. To ensure player confidentiality, all data were anonymized prior to analysis.

### Procedures

**FIGURE 1:**
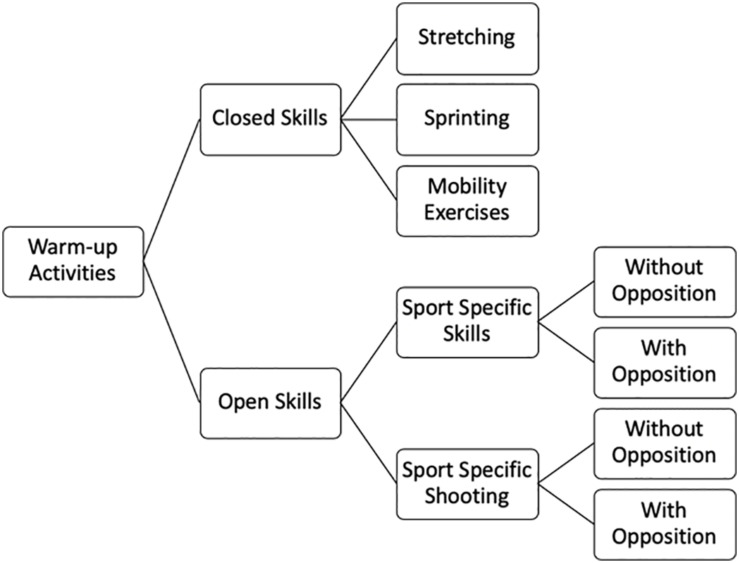
Classification of exercises under specific categories according to their characteristics.

As warm-up routines in futsal included several exercises, they were aggregated under specific categories according to their characteristics (Figure 1). This classification was made by two experts (UEFA A level) with more than 10 years of experience. They individually classified each exercise and if an agreement was not reached, exercises were reviewed and classified accordingly. In particular cases, due to the lack of specific nomenclature reported by literature, the experts’ classification was considered to create specific and reliable categorization. The exercises were classified as (i) Closed Skills—Mobility Exercises (general warm-up exercises of sub-maximal aerobic intensity in order to increase muscle temperature, including jogging, running, skipping forward and backward, and plyometric exercises); (ii) Closed Skills—Stretching (differencing static and dynamic stretching exercises); (iii) Closed Skills—Sprinting (considered by different distances of the sprinting activity after visual or audio signal from the coach with two different areas, i.e., 10- and 20-m distance); (iv) Open Skills—Futsal-Specific Skills without Opposition (technical–tactical exercises without opposition to improve specific technical futsal skills; different passing situations were considered using diverse distances between players and distinguishing exercises with two or three players); (v) Open Skills—Futsal-Specific Skills with Opposition (technical–tactical exercises such as rondos and SSG; exercises were considered according to changes in space and number of players); (vi) Open Skills—Futsal-Specific Shooting without Opposition (individual or two-player combinations for shooting and tactical combinations with three or four players in a progressive pattern till shooting action); and (vii) Open Skills—Futsal-Specific Shooting with Opposition (exercises of counterattack and offensive–defensive transition; different numerical relations were created between attackers and defenders, e.g., 2 × 1 + GK; 3 × 1 + GK; 3 × 2 + GK).

A non-experimental descriptive design was used to record and register the external load of players during warm-up routines before official futsal matches. Players’ activity was assessed using inertial measurement units (IMU) with ultra-wideband tracking system technology (WIMU PRO^TM^, RealTrack Systems, Almeria, Spain). The sampling frequency for the positioning system was 20 Hz. The devices were turned on 10–15 min before the warm-up began. They were placed on players with a specific custom neoprene vest (Svilar et al., 2019). The system has six ultra-wideband antennas, placed 4 m outside the court. The system operates using triangulations between the antennas and the units and derives the unit position (X and Y coordinates) using one of the antennas as a reference. Data were analyzed using SPRO Software (RealTrack Systems, Almeria, Spain). The accuracy and reliability of these devices have been previously reported and validated (Pino-Ortega et al., 2018; Hernandez-Belmonte et al., 2019).

### Descriptive Characteristics

In order to characterize the warm-up routine, the following variables were analyzed: (a) total duration of the warm-up, (b) duration of different categories, (c) number of warm-up categories, (d) sequence of tasks, (e) number of exercises, and (f) exercises used in each category. To analyze the workload demands of warm-up categories, the following external load variables were calculated: total distance covered (m), total distance covered per minute, running distance per minute (12–18 km/h), sprinting distance per minute (>18 km/h), number of accelerations per minute (>2 m/s^2^), and number of decelerations per minute (<−2 m/s^2^).

### Data Analysis

A description of warm-up tasks and its sequence was performed using mean, standard deviations, and percentage of use of each warm-up task in each warm-up and its order over the warm-up. Every variable analyzed through non-parametric comparisons is reported by median and range in the text or in a table.

To characterize the pre-match warm-up workloads, a Shapiro–Wilk test was used to assess the normal distribution of data. Due to the existence of non-normal distribution of data, the differences between warm-up tasks were assessed using the Kruskal–Wallis test and the Dwass-Steel-Critchlow-Fligner to pairwise comparisons. To characterize the warm-up workload profile of each exercise in the same category, individual exercises external load variables were compared with Mann–Whitney *U* test.

Finally, a discriminant analysis was used on the workload indicators (dependent variables) from the warm-up tasks (independent variables) to create a function that classifies the tasks as accurately as possible. Three discriminant functions were obtained and interpreted based on the examination of structure coefficients (SC) greater than | 0.30| (Tabachnick and Fidell, 2007). The statistical specifications of the model included (i) the eigenvalues that show the canonical correlation, whose value (between 0 and 1) indicates to what extent the discriminant variables make it possible to differentiate among the task categories; (ii) Wilks’ Lambda, which expresses the total variability proportion not due to the differences among the task categories; (iii) group centroids that show the location of the task categories in each of the discriminant functions, making it possible to see if they are located, on average, in the positive or negative scores of the function; (iv) the SCs determine the correlation of the variables with the discriminant functions, those of the first function being the ones with the greatest discriminative capacity (the larger the magnitude of the coefficients, the greater the contribution of that variable to the discriminant function, showing the ones that contribute most to discriminating from the value ≥ |0.30|).

Statistical analyses were performed using IBM SPSS for Windows statistics, version 22.0 (IBM Corp., Armonk, NY, United States).

## Results

### Description of Warm-Up Tasks and Its Sequence

The mean warm-up routine duration was 27.5 ± 9.2 min, ranging from 18 to 50 min. Only 20% of warm-up routines lasted 15–20 min. The mean total number of warm-up exercises was 9.3 ± 1.8, and mostly comprising open-skill tasks (80% of total exercises). Open skills were the mostly used, particularly exercises without opposition (more than 50% of the mean time of total warm-up routines). Tasks with opposition (SSG or Counterattacks) were also meaningful. Closed-skill tasks were the least used and were also shorter in duration (see Table 1).

**TABLE 1:** Description of task categories, duration, % of occurrence, and number of exercises in each category.

**Activity**	**Duration (min)**	**Warm-up**	**Exercises (*n*)**
**Closed skills**			
Stretching	2.0 ± 0.67	70%	1.6 ± 0.5
Sprinting	1.1 ± 0.7	80%	1.3 ± 0.5
Mobility exercises	3.5 ± 1.32	30%	3.0 ± 1.7
Sub-total	1.9 ± 1.2		1.7 ± 1.0
**Open skills**			
Futsal-specific skills without opposition	7.4 ± 5.43	90%	2.0 ± 0.9
Futsal-specific skills with opposition	4.9 ± 2.5	100%	1.2 ± 0.4
Futsal-specific shooting without opposition	8.4 ± 2.3	100%	2.6 ± 0.7
Futsal-specific shooting with opposition	4.0 ± 2.01	90%	1.7 ± 0.7
Sub-total	6.2 ± 3.7		1.8 ± 0.9

*Duration – mean duration in minutes of each activity; warm-up – percentage of the presence each activity in the studied warm-up routines; Number of exercises – number of exercises in each category.*

Sport-specific shooting tasks without opposition were the longest exercises with nearly 8.5 min and included three exercises (individual shooting, two-player combinations or set pieces shooting, and tactical combinations). Such exercises were observed in all warm-up routines. The priority given to futsal-specific activities was noticeable because skill activities with and without opposition as well as futsal shooting activities, such as counter attacks, were a key part of the warm-up routine with a mean total of 7.5, 5.0, and 4.0 min, respectively. All of these tasks were observed in 90% of warm-up routines.

The shortest exercises were closed-skill tasks such as stretching and sprinting. Stretching exercises lasted for approximately 2 min. Static stretching was the most used activity with 67% of the total stretching time. Finally, sprinting tasks lasted 1.1 ± 0.7 min, and 90% of this activity was performed in a 10-m space with players responding to acoustic or visual stimulus. In some cases, these sprints were combined with changes of direction.

After analyzing the sequence of tasks, a generic pre-match warm-up routine sequence emerged through the identification of patterns on the use of warm-up categories. Stretching tasks were mainly performed in the beginning of the warm-up. Regarding open-skill tasks, futsal-specific skills were performed before futsal-specific shooting. In both categories, warm-up evolved from tasks without opposition to tasks with opposition (i.e., SSG or counterattacks with different numerical relationships). All sprinting tasks were performed in the final of the warm-up routine (see Table 2).

**TABLE 2:** Sequence and percentage of activities occurred at the beginning, middle, or final phase of the warm-up.

**Activity**	**Sequence**	**Percentage of activity in part of the warm-up**
Stretching	Beginning	57%
	Middle	29%
	Final	14%
Sprinting	Beginning	0%
	Middle	0%
	Final	100%
Futsal-specific skill without opposition	Beginning	100%
	Middle	0%
	Final	0%
Futsal-specific skill with opposition	Beginning	40%
	Middle	60%
	Final	0%
Futsal-specific shooting without opposition	Beginning	0%
	Middle	40%
	Final	60%
Futsal-specific shooting with opposition	Beginning	0%
	Middle	10%
	Final	90%

### Characterization of Warm-Up Workload

**FIGURE 2:**
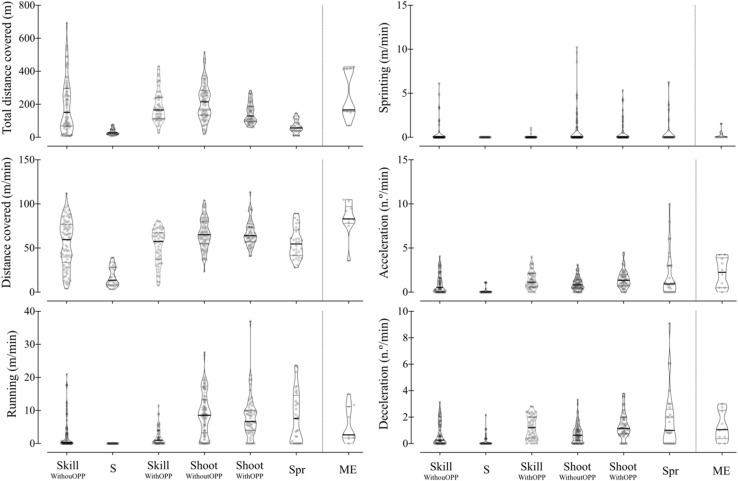
Violin plots of the descriptive analysis. Medium horizontal line represents the median. The lower and upper horizontal lines correspond to the first and third quartiles (the 25th and 75th percentiles). SkillWithouOPP, skill without opposition; S, stretching; SkillWithOPP, skill with opposition; Shoot WithoutOPP, shooting without opposition; ShootWithOPP, shooting with opposition; Spr, sprinting; ME, mobility exercises.

Considering the warm-up categories, mobility exercises revealed the highest amount of distance covered per minute (a median of 83 with a range of 35.6–105 m/min) followed by tasks that involved shooting exercises with or without opposition (65 ranging from 23.4 to 104 m/min, and 63.9 ranging from 40.7 to 113 m/min). Sport-specific skill and sprinting tasks revealed lower values of distance covered per minute than mobility and shooting exercises. The least demanded activity of distance covered per minute was stretching (12.3, ranging from 1.12 to 64.1 m) (see Figure 2).

The running distance per minute achieved the higher values in sport-specific shooting and sprinting tasks, with values ranging from 0 to 27.6 with a median of 8.54 and 0–23.6 with a median of 7.56 m/min, respectively. Sport-specific skill and mobility exercise tasks showed the lowest distances covered. Stretching exercises did not consider any activity that involved higher speed thresholds than running and sprinting.

Tasks with opposition, regardless of being skill- or shooting-oriented, sprinting tasks, and mobility exercises were the most demanding regarding the number of accelerations and decelerations per minute. As verified in other external load variables, stretching exercises were the least demanding. In all tasks, the number of accelerations was higher than decelerations (see Table 3).

**TABLE 3:** Descriptive analysis of each activity.

**Variables**	**Total distance covered (m)**			**Distance covered (m/min)**			**Running (m/min)**			**Sprinting (m/min)**			**Accelerations (n/min)**			**Decelerations (n/min)**		
	**Median**	**Min**	**Max**	**Median**	**Min**	**Max**	**Median**	**Min**	**Max**	**Median**	**Min**	**Max**	**Median**	**Min**	**Max**	**Median**	**Min**	**Max**
Skill_*WithoutOPP*_	231	8.4	431	61.4	10.2	92.8	0.7	0	21	0	0	6.1	0.9	0	4.1	0.6	0	3.1
Stretching	13.4	1.1	68.4	12.3	1.1	64.1	0	0	0	0	0	0	0	0	3	0	0	2.2
Skill_*WithOPP*_	166	26.8	283	57.5	7.7	80.7	0.9	0	11.5	0	0	1.1	1.1	0	4	1.2	0	2.8
Shoot_*WithouOPP*_	215	19.4	517	65	23.4	104	8.5	0	27.6	0	0	10.3	0.8	0	3.1	0.7	0	3.3
Shoot_*WithOPP*_	129	61	283	63.9	40.7	113	6.7	0	37	0	0	5.4	1.3	0	4.5	1.2	0	3.8
Sprinting	56.7	9.2	147	54.6	27.9	89.2	7.6	0	23.6	0	0	6.3	0.9	0	10	1	0	9.1
Mobility exercises	166	71.3	427	83	35.6	105	2.2	0	15	0	0	1.6	2.3	0	4.2	1.1	0	3

Summarizing, mobility exercises, sprinting tasks, and shooting tasks revealed higher values of distance covered per minute at higher speed thresholds than skill tasks as well as the number of accelerations and decelerations per minute. Stretching exercises were the least demanding exercises in all external load variables considered in this study.

### Warm-Up Workload Profile

Looking to the data obtained for all variables, the warm-up model slightly changed between variables through warm-up duration, although an overall consistent trend was still observed. Total distance covered and distance covered per minute had a non-linear profile with the first half of the warm-up having similar distances covered interspersed with the lowest value of the all warm-up and there was a decrease in the final two tasks. When analyzing distance covered at different speed zones, a slight difference was observed between running and sprinting. At the end of the warm-up, shooting exercises with opposition and sprinting tasks showed a lower median value than the previous activity, but with considerable number of players with highest values than in previous activity. Acceleration and deceleration profiles were consistent with an undulatory pattern throughout the duration of the warm-up and ending with a rising number of actions of high intensity.

When applied with a more pronounced analysis, taking into consideration some formal exercise variables such as number of players, space, and dynamics in each category, some key tendencies emerged. Regarding skill without opposition, it was observed that passing exercises with three players revealed significant higher values in sprinting (*p* = 0.011) when compared to two players. Sprinting exercises performed in 20 m demanded a higher running distance from players (*p* = 0.034) in opposition to a lower number of decelerations than 10-m sprinting (*p* = 0.02).

Elite futsal teams used SSG and rondos under the sport-specific skill exercises with opposition. SSG were the most demanding in all running variables (*p* < 0.001 for all variables), with the exception of sprinting distance, while maintaining a coherent profile regarding the number of accelerations and decelerations.

Breaking down sport-specific shooting exercises without opposition, it was possible to identify that tactical combination exercises were most demanding in distance covered at higher thresholds (>18 km/h) (*p* = 0.019 and *p* < 0.001).

Counterattacks were part of 90% of the warm-up routine and coaches used two different strategies with two or three attackers. Three-attacker counterattack exercises (3 × 2 + GK or 3 × 1 + GK) recorded a significant higher number of accelerations and decelerations (*p* = 0.023 and *p* = 0.03, respectively) (see Table 4).

**TABLE 4:** Warm-up load demands profile in each warm-up category compared with Mann–Whitney *U* test.

**Variables**		**Total distance covered (m)**			**Distance covered (m/min)**			**Running (m/min)**			**Sprinting (m/min)**			**Accelerations (n/min)**			**Decelerations (n/min)**		
		**Median**	**Min**	**Max**	**Median**	**Min**	**Max**	**Median**	**Min**	**Max**	**Median**	**Min**	**Max**	**Median**	**Min**	**Max**	**Median**	**Min**	**Max**
Skill_*WithoutOPP*_	Pass 2 ply	193	19	326	52.9	25.4	92.8	0.43	0	11.3	0	0	0	1	0	3.43	1	0	3.14
	Pass 3 ply	253	8.36	693	66.1	10.2	90.5	1.92	0	21	0	0	6.13	0.8	0	4.06	0.29	0	2.4
	*P*	0.129			0.551			0.109			**0.011**			0.67			0.088		
Stretching	Dynamic	26.3	5.8	68.4	17.5	3.5	64.1	0	0	0	0	0	0	0	0	3	0	0	2
	Static	13	1.12	41	8.93	1.12	38.6	0	0	0	0	0	0	0	0	1.09	0	0	2.17
	*P*	**0.035**			**0.002**			n.a.			n.a.			**0.044**			**0.013**		
Skill_*WithOPP*_	SSG	239	109	431	67.3	54.6	80.7	3.54	0	11.5	0	0	1.08	1.41	0	3.33	1.4	0	2.82
	Rondo	140	26.8	277	38.9	7.67	71.3	0	0	8.8	0	0	0.79	0.89	0	4	1.04	0	2.4
	*P*	**<0.001**			**<0.001**			**<0.001**			0.443			0.682			0.329		
Shoot_*WithouOPP*_	Combinations	216	19.4	517	63.2	23.4	104	8.46	0	20	0	0	9.61	0.76	0	3.12	0.6	0	2.41
	Tactical	199	68.7	361	72.6	45.8	99.4	12.7	0	27.6	0.83	0	10.3	1	0	2.67	0.72	0	3.34
	*P*	0.986			0.085			**0.019**			**<0.001**			0.255			0.282		
Shoot_*WithOPP*_	2 attackers	120	61	283	64.8	40.7	113	6.3	0	37	0	0	5.36	1.07	0	3.18	1.06	0	3
	3 attackers	134	83.1	225	63	41.5	83.3	8.39	0	16.1	0	0	4.19	1.81	0	4.5	1.52	0	3.79
	*P*	0.781			0.277			0.598			0.346			**0.023**			**0.03**		
Sprinting	10 m	58.7	9.22	147	58.9	27.9	89.2	3.92	0	23.6	0	0	3.13	1.05	0	10	2	0	9.09
	20 m	56.3	48.7	66.6	48.1	41.6	56.9	16.5	0	23.2	0	0	6.26	0.855	0	0.855	0	0	0.855
	*P*	0.906			0.331			**0.034**			0.136			0.135			**0.02**		

*Pass 2 ply, passing exercises between 2 players; Pass 3 ply, passing exercises between 3 players with movement; SSG, small sided games with different formats; rondo, competitive exercises with one or two defenders with limited movements; combinations, two or three players shooting combinations; tactical, tactical shooting pattern with progression; 2 attackers, counterattack situations with 2 attackers; 3 attackers, counterattack situations with 3 attackers. Bold values represent *p* < 0.05.*

### Dynamics of Each Activity Category

The discriminant analysis revealed differences in the characteristics of each warm-up category. The summary of discriminant functions showed that the first function explained ∼51% of the data variability, while the second explained ∼30% and the third explained ∼16% (see Table 5). Based on the structure coefficients >|0.30|, the first function is determined by all variables, with the exception of number of decelerations, while the second function confirms the high correlation between running and sprinting distance. The first function allows to differentiate the stretching tasks from the other categories due to the low values recorded in all variables followed by skill tasks ending with mobility exercises recording the higher value. The second function discriminates between sprinting tasks and shooting with opposition tasks from the rest of the warm-up categories, highlighting the low values of the discriminant variables of this function, namely, running and sprinting distance per minute.

**TABLE 5:** Summary of discriminant functions.

**Variables**	**Function 1**	**Function 2**	**Function 3**
**Eigenvalues**			
Eigenvalue	0.605	0.360	0.195
% of Variance	50.9	30.2	16.4
Cumulative%	50.9	81.1	97.5
Wilks’ Lambda	0.373	0.598	0.813
Chi-square	374.708	195.200	78.639
Significance	<0.001	<0.001	<0.001
**Structure coefficients**			
Total distance covered (m)	0.937*	0.277	0.137
Distance covered (m/min)	0.674*	−0.188	−0.601*
Running (m/min)	0.406*	0.797*	0.058
Sprinting (m/min)	0.312*	−0.391*	0.557*
Accelerations (n/min)	0.352*	−0.232	0.585*
Decelerations (n/min)	0.193	−0.001	−0.257
**Functions at group centroids**			
Stretching	−2.049	−0.484	−0.464
Sprinting	0.351	−1.496	0.227
Mobility exercises	1.073	0.581	0.984
Sport-specific skill without opposition	−0.336	0.517	0.227
Sport-specific skill with opposition	−0.421	0.270	0.636
Sport-specific shooting without opposition	0.483	0.319	−0.504
Sport-specific shooting with opposition	0.642	−0.517	0.072

*SC discriminant value ≥ |0.30|.

**FIGURE 3:**
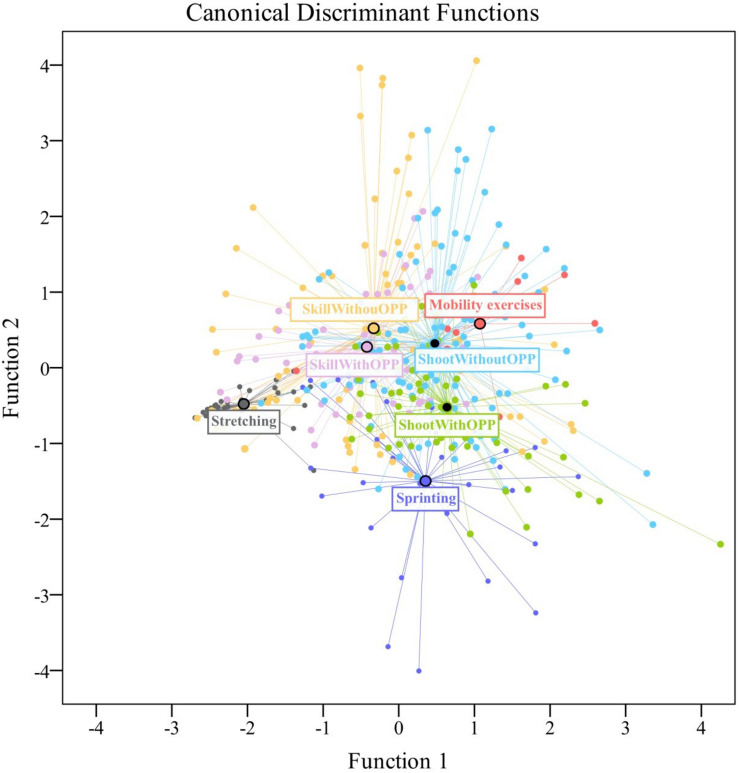
Territorial map of discriminant functions for the task categories of the elite warm-up.

The territorial map of the discriminant functions allows one to observe that closed-skill tasks had a more convergent behavior with a low number of values away from the group centroid than open-skill tasks. Also, skill exercises were more similar to each other than shooting exercises regardless of the existence of opposition (see Figure 3).

## Discussion

This study aimed to identify the dynamics and structure of warm-up routines in elite futsal. From this analysis, type, duration, intensity, sequence, and structure of the tasks emerged as key variables that should be discussed under a combined scope of evidence-based practice and real-world performance enhancement strategies.

Even though natural differences were observed within teams’ WU, a global and coherent warm-up model emerges from the data recorded. Sport-specific skills without opposition and static stretching exercises are globally used as primary warm-up exercises with a total duration closer to 9 1/2 min. Following that, teams tend to prioritize 5 min of sport-specific skill exercises with opposition such as SSG or rondos with different area and numerical relations that precede sport-specific shooting exercises. These routines typically progress from no-opposition (i.e., tactical combinations, individual shooting and set pieces, ≈8 1/2 min) to game-like situations (i.e., counterattacks of different numerical relations between attackers and defenders gradually increasing the number of players, ≈4 min). Repeated sprinting exercises such as 10-m linear accelerations and with changes of direction are systematically used to close WU. This warm-up model lasts for 27 1/2 and includes 11 exercises grouped under different categories with special emphasis on open skills with important ecological value to match performance.

The specific skill tasks displayed important game-related contextualized situations to promote a strong link and transference effect to match performance. Under this scope, coaches designed shooting exercises replicating positional attack patterns, set pieces, and counterattacks that seem to be the most decisive phases of the game in order to score (Sarmento et al., 2018; Mendez et al., 2019). Remarkably, shooting exercises with no opposition are representative of the most important shooting situations in futsal (Sarmento et al., 2016), such as two-player combinations, set pieces shooting, and even specific tactical combinations (Leite, 2012). This ability to replicate offensive situations during positional attack is also enhanced through skill exercises that included key technical actions like passing, receiving, and driving the ball. Skill tasks with opposition are also determinant to create game-like situations where players may improve their adaptability and variability to competitive situations in rondos or SSG (Mendez et al., 2019).

Static stretching was used by the majority of the teams. In fact, it seems to be a common practice in team sports WUs, even though it is an equivocal evidence-based practice. Although static stretching effects on muscle power and strength has been shown to be trivial or even detrimental to subsequent performance (McGowan et al., 2015), the present investigation showed that 67% of the total stretching exercises were dedicated to static routines. It is important to notice that this strategy may compromise acute potentiation, especially if performed for a long period of time (Pojskic et al., 2015a,b; Jamshidi et al., 2016). Even if the use of short periods of static stretching appears to be trivial to subsequent performance (Blazevich et al., 2018), choosing dynamic stretching seems to be a more adequate option because of the higher transfer effect to performance, especially in high-elite athletes (Chaabene et al., 2019).

Sprinting tasks were also frequently used by coaches in warm-up including visual and/or acoustic signals on intermittent sprinting bouts of 10–20 m distance. This option seems to potentiate subsequent sprint performance, particularly when performed at the end of the warm-up (Guinoubi et al., 2015; van den Tillaar and von Heimburg, 2016). These effects may be explained by the PAPE effect as it is expected that high-intensity actions activate temperature and metabolic mechanisms that enhance subsequent performance (Blazevich and Babault, 2019).

It should be expected that these exercises would stand out on external load data; however, exercises with higher sprinting distance covered per minute emerged. It is important to notice that 20-m sprints allowed players to reach a sprinting threshold while 10-m bouts highlighted the number of accelerations and decelerations. Taking into consideration these results, it seems more appropriate to perform sprinting exercises in 10- and 20-m spaces in order to let players achieve the desired sprinting actions without compromising accelerations and decelerations.

Interestingly, data of the present investigation show that the mean total duration of the warm-up was twice that suggested by recent studies, with none of the 10 WUs being performed within the referenced time window. Shorter WUs appear to be more effective for sprint performance enhancement than longer warm-up routines (Yanci et al., 2019). In fact, a recent review advices a total warm-up duration of 10–15 min to have an acute positive effect on muscle temperature and subsequent physical performance (Silva et al., 2018). These results may be particularly relevant due to the necessary balance between potentiation and fatigue induced by warm-up exercises. The desired acute enhancement of player performance is highly associated with the increase of temperature (McGowan et al., 2015) that occurs in the first 3–5 min of warm-up and plateaus after a 10–20-min interval (Bishop, 2003). Not compromising this positive effect is key to performing under the previously mentioned time frame to prevent muscle glycogen depletion and guarantee heat-storage capacity (Bishop, 2003).

After analyzing the duration of different tasks, it is possible to understand that coaches prioritized open skills rather than closed skills. This pattern could be explained by the methodological paradigm of futsal coaches and the positive effects of the use of specific exercises in short-time performance. In the past years, a methodological approach has emerged that integrates all performance factors with warm-up exercises that combine physical demands with a technical and tactical purpose in order to create a pre-match setting with the upcoming match context where the theoretical referential supports this decision; thus, the use of open-skill tasks has shown to be equally effective as closed-skill tasks in key abilities in team sports, namely, futsal, such as sprinting, change of direction, and reactive agility performance (Gabbett et al., 2008). Furthermore, it appears that the use of specific tasks can have an added neuromuscular activation, further ergogenic benefits (Bishop, 2003; Andrade et al., 2015), and high ecological value. However, coaches should be aware that open-skill exercises promote larger individual variability than closed-skill exercises. The chaotic and unpredictable nature of game-like situations where movement patterns and external load applied to players are dependent of all performance factors could drive coaches to have specific closed-skill tasks to ensure that every player reaches an appropriate readiness condition in the end of the warm-up routine.

The warm-up intensity is strongly linked to its total duration and task sequence and structure. It is suggested that warm-up intensity above the anaerobic threshold (i.e., 90% of HRmax and an RPE of at least 16) may have positive effects in sprint performance, possibly up to 10 min (Anderson et al., 2014). An ecological interpretation of warm-up intensity should consider the load imposed to players under the scope of physiological (kinetic energy from distance covered at different speeds) and biomechanical demands (number of accelerations and decelerations) of each activity (Vanrenterghem et al., 2017). Also, warm-up demands should be analyzed under an integrative and ecological approach to evaluate real match context representativeness (Bradley and Ade, 2018). The first pattern that emerges is that shooting exercises are physiologically more demanding than skill exercises, which can be explained by the fact that shooting exercises demand more dynamic actions to progress until the shooting situation that itself involves high-speed actions unlike skill exercises that are designed to highlight technical actions with limited player possibilities. From a biomechanical load standpoint, there is a more balanced profile between all exercises but with three different levels. Exercises without opposition recorded a lower value of accelerations and decelerations than exercises with opposition, independently of their context, highlighting that being in a competitive task can have more influence to the goal of each activity due to high-intensity actions needed to beat the opposition. The number of accelerations is greater than decelerations in all tasks, a pattern that is in contrast to the literature (Harper et al., 2019) but can be explained by the intermittent nature of most warm-up exercises, and this trend could be key to protect players from the negative outcomes associated to decelerations. Closed-skill tasks presented an expected demand from the physiological and biomechanical perspective with sprinting exercises being in the more demanding group and stretching being the less demanding of all tasks.

The sequence of tasks should allow the desired players’ performance enhancement to the upcoming match, but this analysis is not simple due to the scarcity of warm-up studies in team sports with real-world scenarios. It is possible, though, to claim that a warm-up routine should have a progressively intense ending with high-intensity actions such as sprints (Silva et al., 2018) in order to improve neuromuscular readiness. The physiological load has a mixed pattern because the first half of the warm-up shows a slight downward tendency increased by the presence of the stretching activity. In the second half of the warm-up routine, there is the increase of the intensity, but the final minutes are less demanding, which may limit the PAPE effect (Cuenca-Fernandez et al., 2017) that is desired with the high-intensity actions in the end of the WU. The biomechanical load has a more continuous pattern with a crescent demand through the warm-up routine with the exception of the stretching tasks placed in the beginning of the warm-up and the characteristics of the shooting tasks without opposition. However, these loading profiles seem to be more coherent with the literature and coaches should take this into consideration to achieve the desired effect.

Even though in this study tasks with similar features were coupled under the same category, it is important to notice that each category includes different exercises that could impose different demands and modify loading profiles previously analyzed. We must always look through an integrative magnifying glass where players’ actions have a tactical context where they emerge and formal variable manipulation should lead to the desired effect. Under this scope, the area of the task can be manipulated with different results. Twenty-meter sprint tasks have revealed to be physiologically more demanding and should be prioritized with some previous high-intensity actions in order to cause the desired PAPE effect. In the shooting tasks without opposition, tactical combination exercises were most demanding, and these results may be linked to the increased area that players have to explore and consequent higher physiological demands (Sarmento et al., 2018). Additionally, tactical combinations demand that players cover a 20 × 20 m area with the continuity of every phase of the match—construction of offensive actions, finishing situations, and shooting instead of only the last phases that make up shooting combinations. The use of sport-specific skill exercises with opposition, namely, SSG, is a common practice in team sports warm-up routines (McGowan et al., 2015) due to its benefits from the neuromuscular point of view but also for tactical and technical upgrade regarding closed-skill tasks (Dello Iacono et al., 2019). The use of SSG should be prioritized relatively to rondos due to the lower physiological and biomechanical load of these tasks, and these exercises should maintain the increase of players, demands in preparation for the final part of the WU, but without compromising total duration of warm-up (Zois et al., 2011).

When confronting data recorded from elite futsal warm-up routines with the existing literature, important information emerges that may be carefully analyzed and interpreted in order to build key practical applications for coaches and practitioners. Coaches should be aware that shorter WUs with progressive increased intensity may be optimum to potentiate acute performance. For that purpose, coaches should review the abusive use of static stretching, opting for dynamic and sprinting exercises that should allow players to increase the number of high-intensity running in the final moments of the WU. The balance between open- and closed-skill exercises must be achieved, prioritizing open specific skill exercises that create optimal pre-match contexts but with specific closed-skill exercises that are key in reducing the inter-variability of the load profile applied to players. Additionally, open-skill tasks may be performed under different structures, prioritizing SSG over rondos in the beginning of the warm-up routine, progressing from simple shooting exercises to tactical combinations and three-player counterattacks in order to increase physiological and biomechanical loading.

Future research should analyze the effects of manipulating the aforementioned variables on short-term performance and readiness compared to the warm-up model of elite futsal teams.

## Conclusion

The warm-up model routine that emerged from the present investigation pointed out some issues that should be analyzed and discussed under the scope of real-world practices and the state of the art provided by relevant research. The duration of the warm-up was found to be longer than that reported and advised in research that focused on acute performance enhancement following team sports warm-up, which may impact muscle temperature and performance-related variables such as sprint. The sequence of the tasks also raised some questions because physiological load did not have a clear growing intensity and coaches should be aware that activities with high-intensity actions should be placed in the final part of the warm-up routine to ensure the desired acute performance enhancement. Dynamic stretching exercises should be prioritized instead of the static stretching exercises due to the higher transfer effect and should appear in the final phase of the warm-up routine instead of the beginning. The end of the warm-up was dedicated to sprinting tasks, as advised, but the formal variables of these tasks, namely, the limited space they occur, inhibited players to achieve the intended actions, namely, sprinting distance. Apart from these issues, warm-up routines highlighted the value of sport-specific tasks with exercises representative of the most important actions of a futsal match creating ideal pre-match settings adapted by players for the upcoming game. Futsal coaches should be aware of how to manipulate the formal variables of the tasks to allow players to have the correct actions at the correct time under an integrative approach to the physical continuum that should be an elite warm-up routine.

## Data Availability Statement

The raw data supporting the conclusions of this article will be made available by the authors, without undue reservation.

## Ethics Statement

The studies involving human participants were reviewed and approved by the Ethics Committee of Universidade da Beira Interior (CE-UBI-Pj-2018-029). The patients/participants provided their written informed consent to participate in this study.

## Author Contributions

NS, BT, and EA contributed equally to all, including data collection and data analysis. BG contributed to design, data analysis, discussion, tables, and figures. JB contributed to introduction, data analysis, and results. All authors contributed to the article and approved the submitted version.

## Conflict of Interest

The authors declare that the research was conducted in the absence of any commercial or financial relationships that could be construed as a potential conflict of interest.
